# Fractionated stereotactic radiotherapy for pediatric patients with retinoblastoma

**DOI:** 10.1120/jacmp.v7i2.2161

**Published:** 2006-05-25

**Authors:** Patrick D. Higgins, Bruce J. Gerbi, Mark Macedon, Kathryn E. Dusenbery

**Affiliations:** ^1^ University of Minnesota Department of Therapeutic Radiology‐Radiation Oncology University of Minnesota Minneapolis Minnesota; ^2^ University of Minnesota Marshfield Clinic Marshfield Wisconsin U.S.A.

**Keywords:** pediatric radiotherapy, stereotactic radiotherapy, patient fixation

## Abstract

In this report, we discuss the application of a modified Gill‐Thomas‐Cosman (GTC) relocatable head frame to enable fractionated stereotactic radiotherapy (SRT) of infants under anesthesia. This system has been used to treat two infants, ages 12 and 18 months, for bilateral retinoblastoma on a Varian 6/100 linear accelerator. The GTC head frame was used to reproducibly position and treat the orbits of these children to between 2520 cGy and 3960 cGy in 180‐cGy fractions. A standard head and neck tray, with accompanying thermoplastic mask, was adapted to mount to the head frame to enable these treatments. We found the maximum average deviation in the repeat fixations, as compared with the initial fitting data, to be ±2mm. The overall average difference and standard deviation in measurement was 0.47±0.63mm for the first case and 0.19±0.94mm for the second case, with a combined average of 0.35±0.79mm overall from a total of 381 point measurements. The stereotactic treatment plan (Radionics®) incorporated a single isocenter for each orbit and 3 or 4 arcs per isocenter. An intercomparison has been made between this technique and a standard lateral field technique, designed using the stereotactic radiosurgery (SRS) planning system. Dose‐volume histograms and corresponding normal tissue complication probabilities (NTCP) based on pediatric bone growth inhibition have been calculated for each method for the orbital bone areas. We found that the NTCP is reduced from 95% or more in the standard treatment method to 16% or less with SRT. Use of the modified head frame provides excellent setup reproducibility, facilitates access to patients for anesthesia, and reduces the chances of a poor cosmetic result in these growing children.

PACS number: 87.53.Ly

## I. INTRODUCTION

Retinoblastoma is an uncommon disease that affects 600 children per year in the United States. It is the most common orbital tumor in children and accounts for 5% of childhood blindness. Usually manifesting at a very early age (95% diagnosed before age 5), it is unilateral in 2/3 of patients and bilateral in 1/3 of patients. Bilateral disease is nearly always a sign of a hereditary mutation, while the unilateral disease is more commonly sporadic.

The treatment of intracranial tumors in children poses several unique challenges that require a different approach from adults. Children may be more prone to develop certain specific adverse sequelae as a result of radiation therapy. Neuropsychological sequelae such as memory problems and learning disabilities have been observed.^(1)^ Endocrine dysfunction unrelated to underlying malignancy may also be seen.^(1)^ Second malignancies are also a concern because patients successfully treated as children survive well into adulthood.^(1)^ Finally, radiation to developing bones may stunt growth, resulting in possible facial deformities.^(2)^ In an effort to limit long‐term adverse effects by minimizing bone dose, wherever possible, clinicians have increasingly been turning to more conformal methods of delivering radiation, including stereotactic radiosurgery (SRS), in which large doses (10 Gy to 20 Gy) are delivered in a single treatment, and stereotactic radiotherapy (SRT), which more closely follows conventional radiation therapy, where a course of treatment is fractionated into a number of daily treatments (1.5 to 2.5 Gy/fraction). Proton therapy has also been explored as a treatment option, along with chemotherapy, thermo‐chemotherapy, hyperthermia, and photocoagulation.^(3)^

Compared with single‐fraction SRS, which is the only possible method of treatment on Gamma Knife—type treatment units, fractionated stereotactic radiation therapy also has a considerable advantage for the treatment of intracranial tumors in children. As with conventional radiation therapy, daily fractionation is better tolerated by normal tissue structures.^(^ ^1^ ^,^ ^4^ ^)^ In addition, repeat fixation devices are much less traumatic than the invasive frames required for the SRS method and may be removed much more quickly, if the need arises.

The delivery of fractionated stereotactic treatment in young children poses additional challenges. Small children are unable to remain motionless during radiation therapy and thus require anesthesia. Most conventional immobilization techniques requiring both patient cooperation and teeth (for bite block systems) cannot be used in their standard configuration. In this report, we detail a simple modification that may be applied to a standard fixation device that circumvents these problems and allows us to take advantage of this powerful technique for the treatment of infants.

The most common noninvasive frames for SRT are the Gill‐Thomas‐Cosman (GTC) frame (used here at the University of Minnesota) and the Laitinen system (LS) frame,^(^ ^5^ ^,^ ^6^ ^)^ although variants also exist. The original GTC frame of Gill et al.^(7)^ modified by Kooy et al.^(8)^ uses a rigid ring, split to allow an opening for patient access. Attached to the ring, as an assembly, is a patient‐specific oral appliance (dental plate) custom‐fitted to the upper teeth and palate of the patient. A head support plate (back plate) is attached to the back of the ring and filled with a quick‐setting compound, which is formed to the patient's occiput, providing both support while the patient lies down and additional immobilization. A three‐point Velcro strap is attached to the lateral sides of the ring and the back plate. Pulling this tightly together is the final immobilization step. The ring and immobilization assembly is finally attached to a flange, which is connected to the end of the CT or treatment couch. Variants of these frames have been used for pediatric cases, specifically, a smaller version of the LS frame and an adaptation of the GTC frame at the Boston Children's Hospital (BCH), also reviewed by Kooy et al.^(8)^ The BCH frame uses some of the features of the LS frame system and replaces the oral immobilization appliance with earplugs and a thermoplastic mask, which includes only the anterior face (nose, orbits, forehead, and temples). The BCH frame allows anesthesia access but is limited in the sense that a significant amount of material is added around the cranium, which may reduce access or otherwise affect the dosimetry of retinoblastoma treatment.

Confirmation of position is performed using a depth confirmation helmet. Reproducibility of setup has been satisfactory with Kooy et al.^(8)^ reporting on data from 20 patients a mean measurement error of 0.71±0.06mm (range 0.31 mm to 1.22 mm), mean lateral movement of 0.35±0.06mm (range 0.07 mm to 0.79 mm), mean superior movement of 0.52±0.09mm (range 0.00 mm to 0.94 mm with one outlier at 1.77 mm), and mean occipital movement of 0.34±0.09mm (range 0.00 mm to 1.30 mm). More recently, Burton et al.^(9)^ reviewed the value of using the depth helmet to evaluate repositioning accuracy.

Of two patients treated in our clinic with fractionated stereotactic radiotherapy, one was 18 months old; he presented at age 6 months with decreased red reflexes in both eyes. He was diagnosed with bilateral retinoblastoma and subsequently underwent several cycles of chemotherapy and cryotherapy. He was referred to our clinic when he developed a recurrence in his left retina only months after completing chemotherapy. The tumor in the right eye remained quiescent, but the decision to treat both eyes stereotactically was made. Virtually the same clinical scenario was true for our second patient (12 months old) with bilateral retinoblastoma. For each patient, we used our modified GTC frame. We discuss that construction as well as the quality of positioning accuracy and reproducibility in the following.

To further review the advantages of SRT over conventional treatment techniques for pediatric bilateral orbit treatment, we constructed and analyzed dose‐volume histograms for the orbital bones, comparing SRT with simulated, parallel‐opposed lateral fields. There are more than a few ways of designing bone‐sparing treatment fields beyond a simple bilateral technique. For example, wedged pairs (for single orbits) and 3D‐conformal field designs would be expected to better reduce bone dose.^(3)^ For the SRT case, there is certainly no question that penumbra is much better limited, even if 3D or intensity‐modulated radiotherapy approaches are used for comparison. We originally planned to compare a variety of techniques, but would have had to use different planning systems, requiring the targets and bones to be redrawn and dose‐volume analyses to be, potentially, slightly different between them. By doing all the planning on the same treatment‐planning system (Radionics®), we felt the comparison would be the most self‐consistent. Further, the comparison demonstrates access to advanced 3D treatment planning using this immobilization system.

## II. METHODS

We began by adapting a commercially available head and neck tray (Thermoplastic Masks, MedTec, Orange City, IA) to the GTC ring by simply cutting it to size and screwing it onto the two rear stanchions that normally hold the GTC back plate. These trays are made in‐house but are available commercially, in slightly different forms. They typically include a machined slot with at least two mounting pins for head rests and additional pins and clamps for guiding and retaining Aquaplast® immobilization masks. The dental fixation plate and Velcro straps from the original design are not needed.

**Figure 1:**
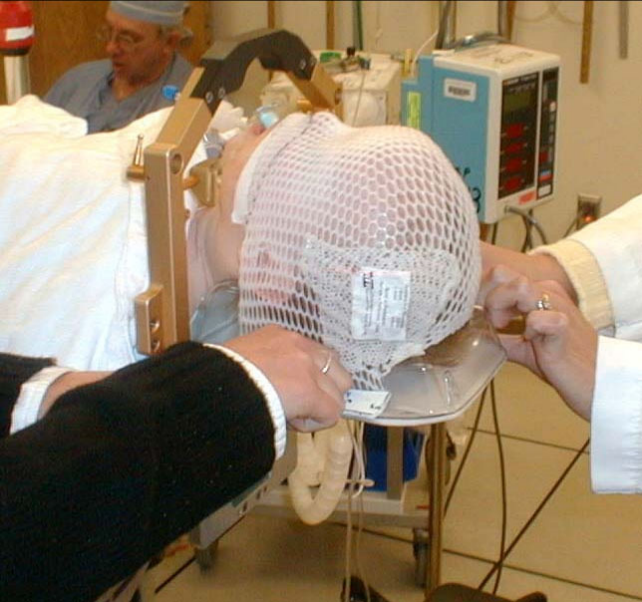
Initial setup. GTC ring (closed) with thermoplast mask being screwed into the baseplate. This combination is the primary difference with the BCH design.

For the two patients treated we used a 1/4‐in. plastic (acrylic) plate. A more rigid plate, such as one made with stainless steel or aluminum, could also be used. In these instances, it was determined that the infants’ small size and weight did not incur significant flexing of the plastic. The ring and support plate are attached to the end of the simulation room table using a standard bracket. The ring assembly may be split above the mounting bracket by releasing two screws and sliding the ring upward along the revealed 4‐in. to 5‐in. long guide pins. With the ring open, the patient may be inserted through the ring and the head cradled in a selected, angled head support. The ring is then closed and secured. A thin, thermoplastic mask, which has the property of being highly elastic when warm and rigid on cooling, is stretched over the upper half of the face and attached to the plate with nylon wing nuts (Fig. 1). Care is taken to open the mesh over the patient's tragi and fleshy canthus to allow a clear view of these anatomical structures to aid patient positioning. A measurement is taken from the nasion to the edge of the ring to fix forward—backward tilt. Right and left lateral level and alignment marks are also made, aligned with the tragi, and checked prior to each treatment with the lateral treatment room alignment lasers. An advantage of this system over the BCH frame is that the immobilizing mask is thin, it is standard for any department, and it is simply attached to a standard, albeit modified, baseplate without forfeiting anesthesia access.

**Figure 2:**
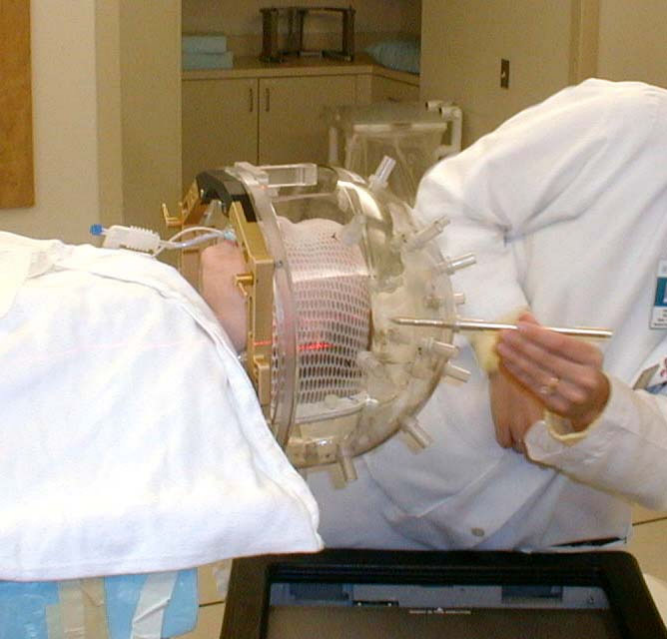
Depth helmet verification step. Depth helmet shown attached to GTC ring. Measurements are taken using a depth gauge of the skin‐to‐helmet distances.

**Figure 3:**
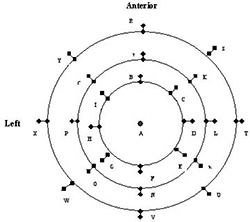
Display of depth helmet geometry as seen from directly behind the helmet.

A key component of the GTC repeat fixation system is a secondary method for evaluating the accuracy of repositioning.^(9)^ This is accomplished using a Radionics® depth confirmation helmet (Fig. 2). This helmet mounts to the base ring and includes 25 holes spaced into three rings. A posterior view, showing the locations of the holes for comparison with Fig. 2, is given in Fig. 3. A depth gauge having millimeter markings is used to measure the depths from the top of each hole to the patient's skin (mask in some instances). These depths provide an aid in evaluating repositioning problems prior to each treatment. Discrepancies of more than 2 mm are reviewed and may require that the patient be repositioned.

**Figure 4:**
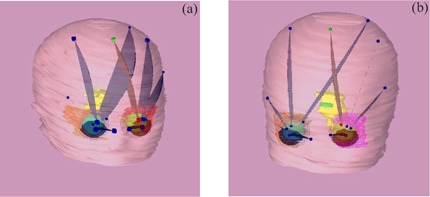
Stereotactic arc arrangement for patients 1 (a) and 2 (b).

**Figure 5:**
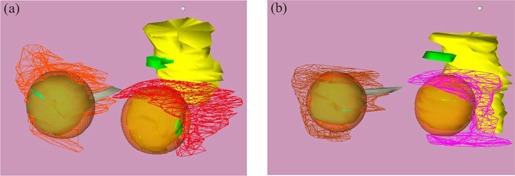
Isodose volumes (shown as orange, transparent surfaces) for 90% isodose (prescription isodose) for patients 1 (a) and 2 (b).

Following the initial fitting, patients receive a CT scan. (The frame is not MRI compatible.) A fiducial marker cage is attached to the base ring, and a series of 2mm×2mm axial scans is acquired through the entire head. Treatment planning is performed using the Radionics® X‐Knife 4™ software. In both preliminary cases, bilateral orbits and disease extension (with 4‐mm to 5‐mm margins) were treated using single isocenters for each orbit and 3.0‐cm to 3.5cm diameter inserts. Three or four arcs (approximately 300° total arc angle) were delivered to each orbit. Figure 4 displays the arc arrangement for patients 1 (Fig. 4(a)) and 2 (Fig. 4(b)). The corresponding dose distributions in which we see the prescription isodose volumes (90%) (orange, transparent region) are shown in Fig. 5.

The first patient (Figs. 1 and 2) (18 months old) received 3600 cGy in 20 fractions over 26 days to his right eye and 2520 cGy in 14 fractions over 18 days to his left. The second patient (12 months old) received 3960 cGy in 22 fractions over 28 days to her right eye and 3600 cGy in 20 fractions over 26 days to her left.

**Figure 6:**
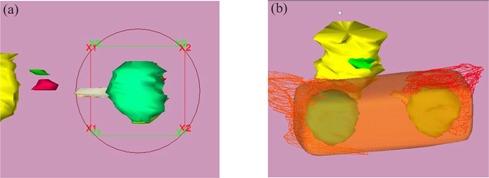
Right‐lateral beam's‐eye view of patient 1 lateral field (a) and 90% isodose volume (b).

The Radionics® X‐Knife 4™ software was also used to simulate a parallel‐opposed lateral treatment technique. To accomplish this, 1° right and left lateral arcs were set up and centered at midline, between the orbits. Five‐centimeter diameter cutouts were shaped, using asymmetric jaws, into rectangular fields having a margin of approximately 1 cm around the indicated targets. Figure 6(a) displays a typical beam's‐eye view of the lateral fields, and Fig. 6(b) the resultant 90% volume dose distribution.

Dose‐volume histograms were constructed for each treatment method. The effective volume method of Kutcher et al.^(10)^ was used to generate effective doses and volumes that apply Lyman's^(11)^ theory with Burman et al.'s^(12)^ parameterization to calculate normal tissue complication probabilities (NTCP). Classically, this model is written as follows:

$$\text{NTCP}=\frac{1}{\sqrt{2\pi }}\int_{-\infty }^{t}\exp\left(\frac{-t^{2}}{2}\right)dt,$$

where, for effective volume $v,\;t=(D-{\text{TD}}_{50}(v))/(m\times {\text{TD}}_{50}(v))$ and ${\text{TD}}_{50}(v)={\text{TD}}_{50}(1)\times v^{-n}$

The parameters *m* and *n* were estimated to be 0.1 and 0.25, respectively, consistent with typical values obtained by Burman et al. The 50% probability of bone growth arrest $\left({\text{TD}}_{50}\right)$, used in the calculation, was estimated as 30 Gy, based on Eifel et al.'s^(13)^ assessment of complication probabilities in growing bone. They noted that the region of steepest dose effect was 15 Gy to 30 Gy, with little significant growth abnormalities reported below 25 Gy.

## III. RESULTS AND DISCUSSION

**Figure 7:**
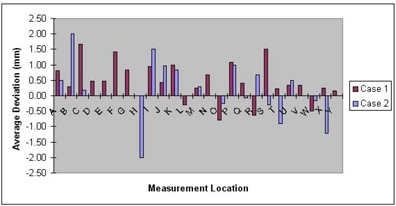
Summary graph of the average setup variations on a point‐by‐point basis, as assayed with the depth helmet, for locations mapped out in Fig. 3.

Each of the two patients received between 20 and 22 total treatments. During this time, positioning accuracy was monitored by performing measurements using the depth helmet before each treatment. Using the initial setup measurements taken prior to CT data acquisition as our baseline, we have computed the deviations between those measured depths and those taken each day prior to treatment. In Fig. 7 we have summarized those measurements in graphical form as the average deviation from baseline as a function of location (see Fig. 3). We found the maximum average deviation to be ±2mm (locations B and H), due to difficulty in obtaining depths to the skin (through the mask) at these locations. The overall errors averaged to 0.47±0.63mm for the first case, 0.19±0.94mm for the second case, and 0.35±0.79mm overall from a total of 381 point measurements. Not all points were sampled before each treatment, with an average of 11/25 checkpoints taken for the first patient and 7.5/25 for the second patient. It was felt that these randomly distributed points provided adequate sampling of the setup precision.

**Figure 8:**
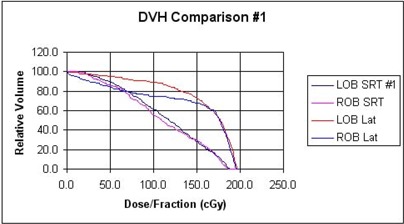
Dose‐volume histogram for SRT and right‐left lateral field treatments of patient 1. LOB and ROB indicated the left orbital bone and right orbital bone, respectively.

**Figure 9:**
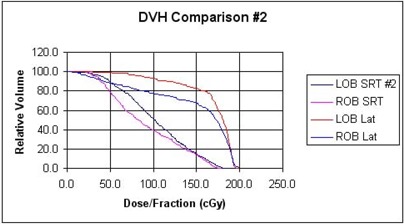
Dose‐volume histogram for SRT and right‐left lateral field treatments of patient 2. LOB and ROB indicated the left orbital bone and right orbital bone, respectively.

Results of the dose‐volume reduction analyses are summarized in Table 1 (with the corresponding dose‐volume histograms displayed in figures 8 and 9) for our two patients and for the compared treatment methods. We find that for the single orbit, treated to 25.2 Gy, both techniques predict less than about 2% complication probability. For orbits treated to 36 Gy, the SRT technique keeps complication probability to 3% or less for both patients, while the lateral technique is predicted to yield between 87% and 95% chance of bone growth arrest. For the single orbit treated to 39.6 Gy, use of SRT results in an NTCP prediction of 6.6% and the lateral field method almost 99%. Presumably, other 3D treatment techniques offer intermediate levels of improvement over the simple lateral technique.

**Table 1:** Comparison of calculated normal tissue complication probabilities for stereotactic versus bilateral radiation techniques

Patient		Right orbital bone	Left orbital bone
#1	Prescription dose	36 Gy	25.2 Gy
	NTCP: SRT	1.6%	0.0%
	R/L laterals	87.5%	2.2%
#2	Prescription dose	39.6 Gy	36.0 Gy
	NTCP: SRT	6.6%	3.0%
	R/L laterals	98.6%	95.3%

## IV. CONCLUSIONS

We have developed a modification of the Gill‐Thomas‐Cosman relocatable head frame that is compatible with anesthesia needs and suitable for treatment of infants of about 3 years old or less. We have found patient setup to be simple and reproducible, with repositioning accuracy of the order of ±1mm or better on average. The modified immobilization system uses a thermoplastic mask that is thinner than that used for the BCH frame and a simple attachment system adapted from a standard head and neck device. We believe that intracranial radiation treatment, particularly of retinoblastoma, is the method of choice for pediatric cases, where sparing of bone growth centers is extremely important for the long‐term normal development of the child.

This immobilization system allows the choice of a variety of treatment approaches including SRT, as well as 3D conformal therapy and intensity‐modulated radiotherapy. We have, as an example, demonstrated the order of gain that can be achieved using fractionated stereotactic radiosurgery versus a simple, parallel‐opposed lateral field technique for bilateral orbit irradiation. Using dose‐volume histogram reduction, we illustrated the kinds of differences to be expected and the level of dose sparing one might hope to achieve with SRT capability. The simplicity of this head frame modification extends stereotactic treatment to pediatric cases that might otherwise have more limited treatment options.

## ACKNOWLEDGMENTS

We would like to thank our chief therapist, Kristin Robinson, and departmental engineer, Roy Erickson, for their contributions to this project.
